# Tungsten Trioxide as a Visible Light Photocatalyst for Volatile Organic Carbon Removal

**DOI:** 10.3390/molecules191117747

**Published:** 2014-10-31

**Authors:** Yossy Wicaksana, Sanly Liu, Jason Scott, Rose Amal

**Affiliations:** Particles and Catalysis Research Group, School of Chemical Engineering, The University of New South Wales, Sydney, NSW 2052, Australia; E-Mails: yossywicaksana@gmail.com (Y.W.); jason.scott@unsw.edu.au (J.S.); r.amal@unsw.edu.au (R.A.)

**Keywords:** tungsten oxide, hydrothermal, photocatalytic, visible light, platinum, photodeposition, ethylene

## Abstract

Tungsten trioxide (WO_3_) has been demonstrated to possess visible light photoactivity and presents a means of overcoming the UV-light dependence of photocatalysts, such as titanium dioxide. In this study, WO_3_ nanostructures have been synthesised by a hydrothermal method using sodium tungstate (Na_2_WO_4_·2H_2_O), sulphate precursors and pH as structure-directing agents and parameters, respectively. By altering the concentration of the sulphate precursors and pH, it was shown that different morphologies and phases of WO_3_ can be achieved. The effect of the morphology of the final WO_3_ product on the visible light photoactivity of ethylene degradation in the gas phase was investigated. In addition, platinum (Pt) was photodeposited on the WO_3_ structures with various morphologies to enhance the photocatalytic properties. It was found that the photocatalytic properties of the WO_3_ samples greatly depend on their morphology, chemical composition and surface modification. WO_3_ with a cuboid morphology exhibited the highest visible light photoactivity compared to other morphologies, while adding Pt to the surface improved the performance of certain WO_3_ structures.

## 1. Introduction

Volatile organic compounds (VOCs) are the group of airborne organic compounds capable of damaging human health and the environment. Indoor environments have been reported to contain up to ten times greater VOC pollutant levels than outdoor environments [1]. Indoor VOCs can originate from building materials, paints, glues, lacquer, carpet, office furnishings, cleaning compounds, cigarette smoke and other items [2,3]. Increasingly stringent regulations regarding acceptable VOC concentrations necessitate the implementation of technologies capable of meeting these requirements. Technologies based on adsorption or scrubbing the gas streams are capable of removing the VOCs, but generate secondary waste streams, which require disposal. Photocatalysis is an alternative technology for treating VOCs, converting them into comparatively benign water and carbon dioxide [4].

Titanium dioxide (TiO_2_) is generally the semiconductor of choice due to its high photoactivity, low cost, ready availability and non-toxic properties. However, TiO_2_ can only be activated by ultra-violet (UV) light (λ ≤ 380 nm), limiting the use of sunlight as the light source and rendering it virtually unusable in indoor environments without the presence of an external UV light source [5]. Tungsten trioxide (WO_3_) is a comparatively less studied semiconductor, which is capable of being activated by visible light (λ ≤ 450 nm) [6] and, consequently, may be a more suitable semiconductor for degrading VOCs in an indoor environment.

Several methods, including chemical vapour deposition (CVD) [7], thermal evaporation [8], electrochemical techniques [9], a spray pyrolysis approach [10], template-mediated synthesis [11], the sol-gel process [12] and hydrothermal reactions [13], have been reported for WO_3_ nanostructure synthesis. Of the listed methods, hydrothermal synthesis is a facile technique, which is well-suited to producing a range of nanostructured morphologies by simple variations to the precursor solution. For instance, controlled synthesis of WO_3_ nanostructures by the hydrothermal method has been performed with the help of structure-directing chemicals, like Na_2_SO_4_, Rb_2_SO_4_, K_2_SO_4_, Li_2_SO_4_, FeSO_4_ and Na_2_S [14,15,16,17,18]. This work focuses on fabricating tungsten oxide photocatalysts using a hydrothermal technique with varying pH, as well as the amount and type of sulphate precursor. Emphasis is placed on the influence of preparation conditions on the characteristics of WO_3_ nanostructures and, subsequently, on its capacity to photodegrade gas-phase ethylene using visible light as the energy source.

The relative energy of the electrons in the WO_3_ conduction band restricts their capacity to reduce oxygen, which results in a build-up of these electrons, followed by an increased incidence of recombination with holes and, ultimately, a decrease in photocatalytic performance. Improvements in WO_3_ photoactivity, so as to counteract this limitation, may be achieved by closely controlling particle morphology [19] or by loading platinum (Pt) deposits on the surface. The noble metal Pt is believed to greatly assist in electron transfer during the oxidation-reduction reactions in the photocatalysis process [20]. Pt loaded onto WO_3_ nanoparticles has been shown to enhance aqueous acetic acid mineralisation compared to bare WO_3_ photocatalysts under visible light illumination [21]. Similarly, WO_3_ photocatalysts with different platinum loadings have been proven to be more efficient for phenol oxidation [22], as well as tetracycline oxidation [23] in an aqueous suspension. Even at a 0.1% Pt loading on WO_3_ nanoparticles, notable improvement of the water splitting reaction has been demonstrated [24]. A beneficial impact of Pt deposits on WO_3_ was also observed in the gas phase for acetaldehyde photodegradation [25]. In this work, the influence of loading the surface with nanosized Pt deposits on visible light photoactivity is also considered.

## 2. Results and Discussion

The alkaline solution precursor of Na_2_WO_4_·2H_2_O essentially contains stable WO_4_^2−^ ions. With the supply of H^+^ ions from the resin, the WO_4_^2−^ ions gradually underwent condensation reactions in the column to form paratungstate ions, such as [W_12_O_41_]^10−^ and [H_2_W_12_O_40_]^6−^ (at pH ~ 4.0–7.0), and the metatungstic acid, (WO_3_)_n_·xH_2_O, (at pH ~ 1–4), according to the equations below [26].

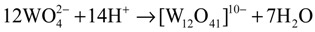
(1)

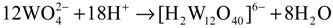
(2)

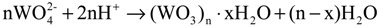
(3)

The condensation reaction involves protonation of tungsten oxyanions, formation of oxygen bridging between the protonated monomeric tungsten oxyanions and the release of water molecules. The rates of condensation are strictly controlled by the flow rate of solution and the amount of resin used.

**Figure 1:**
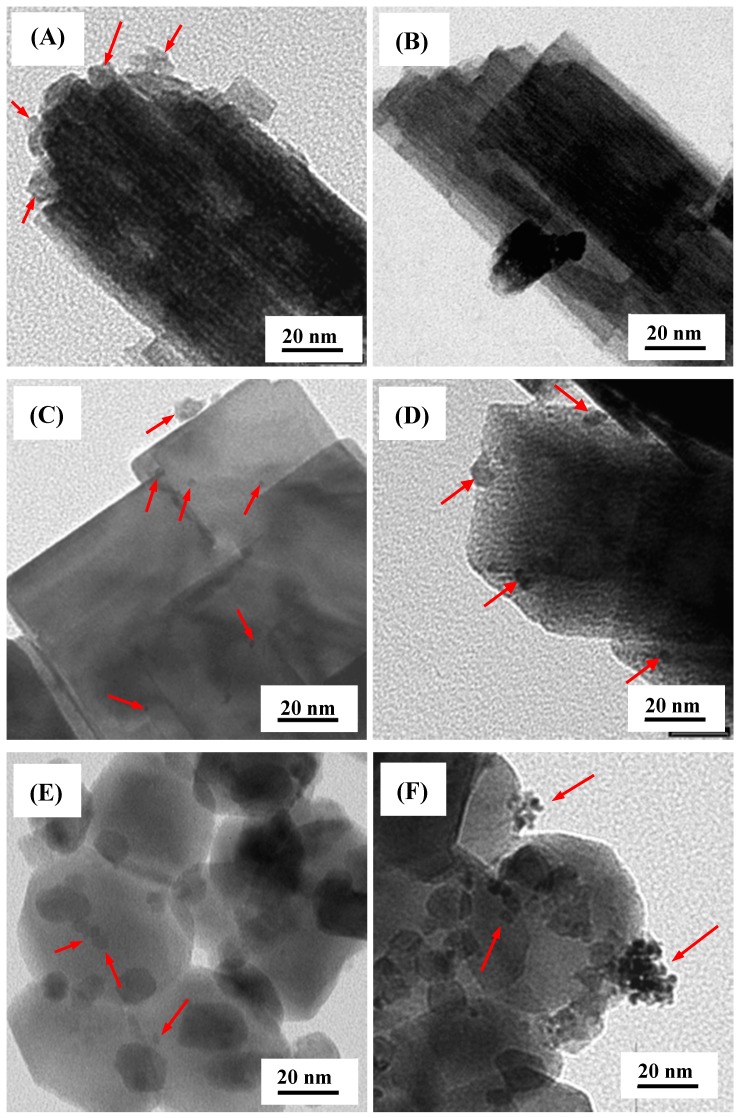
SEM images of: (**A**) hydrothermally synthesised WO_3_ with no additives (W0); (**B**) hydrothermally synthesised WO_3_ with Na_2_SO_4_ added at a SO_4_^2−^:WO_4_^2−^ ratio of 0.3 (W0.3NaS); (**C**) hydrothermally synthesised WO_3_ with Na_2_SO_4_ added at a SO_4_^2−^:WO_4_^2−^ ratio of 7.6 (W7.6NaS); (**D**) hydrothermally synthesised WO_3_ with H_2_SO_4_ added at a SO_4_^2−^:WO_4_^2−^ ratio of 0.3 (W0.3HS); (**E**) hydrothermally synthesised WO_3_ with H_2_SO_4_ added at a SO_4_^2−^:WO_4_^2−^ ratio of 7.6 (W7.6HS); (F) commercial Sigma Aldrich WO_3_ (WSA).

WO_3_ particles prepared from the neat tungstic acid precursor (*i.e*., W0) were found from ICP-AES analysis to contain a sodium atomic content of 2.78 × 10^−4^ per atom of W. Since the starting solution contained two atoms of Na per atom of W, the significant reduction from the original amount indicates high removal efficiency by the ion-exchange process. The W0 particles were yellow in colour and identified by XRD analysis (spectra are located in the Supporting Information, Figure S1) to be a mixture of the monoclinic WO_3_ phase (cell constants: a = 7.2970 Å, b =7.5390 Å, c = 7.6880 Å; JCPDS 01-071-2141) and the orthorhombic WO_3_·⅓H_2_O phase (cell constants: a = 7.3590 Å, b = 12.5130 Å, c = 7.7040 Å; JCPDS 00-035-0270). The mixture of phases in W0 conforms to the tungstic acid dehydration evolution profile as described by Livage and Guzman [27]. SEM imaging indicates that the W0 morphology comprises a mixture of large slabs with clusters of columnar crystals, as observed from Figure 1A.

Figure 1F shows the SEM image for the commercial Sigma Aldrich WO_3_ (WSA). WSA consists of nanoparticles with a size distribution from 30–100 nm and, from XRD (spectra are located in the Supporting Information, Figure S1) was found to possess a pure monoclinic crystalline phase.

### 2.1. Effect of Sulphate Anions and pH on WO_3_ Characteristics

The impact of sulphate (originating from Na_2_SO_4_) as the shape directing agent on WO_3_ morphology is illustrated in Figure 1B,C for W0.3NaS and W7.6NaS (see Experimental Section 3.2 and Table 1 for an explanation of the sample abbreviations), respectively. The two samples show more elongated structures when compared to W0. The W0.3NaS particles consist mainly of randomly orientated columnar crystals and individual particles, while the W7.6NaS particles exhibit a bundled structure consisting of aligned nanorods. The difference in the morphology can be attributed predominantly to the addition of the SO_4_^2−^ anions, as the pH values of the final solutions were similar (Table 1). It is readily apparent that the SO_4_^2−^ anions promote anisotropic growth. One manner by which shape-controlling additives can behave is to “cap” the growth of particles along a particular crystal plane. That is, they preferentially interact with one or two crystal faces, hindering growth on those planes, which then favours growth on the “uncapped” face, resulting in elongated structures [28]. To explain the tendency of the nanorods to align parallel to each other for W7.6NaS, it is speculated that the lateral capillary forces along the length of the nanorod are higher compared to its width, causing side-by-side alignment rather than end-to-end. The aggregation of the pre-formed nanorods by oriented attachment [29] may be energetically favoured to reduce the surface energy of the system, and consequently, the nanorod WO_3_ bundles are obtained for W7.6NaS.

**Table 1:** Selected synthesis parameters and characteristics for the different WO_3_ photocatalysts. Included are the characteristics for commercial Sigma Aldrich WO_3_ (WSA).

Sample	Precursor Solution	Molar Ratio 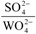	Final pH	Morphology	Crystalline Phase (Chemical Formula)	Surface Area (m^2^/g)	Band-Gap (eV)
W0	20 mL 0.1 M H_2_WO_4_	-	1.61	slabs with columnar clusters	monoclinic (WO_3_) + orthorhombic (WO_3_·⅓H_2_O)	n.a.	n.a.
W0.3NaS	20 mL 0.1 M H_2_WO_4_ + SO_4_^2−^ (from Na_2_SO_4_)	0.3	1.3	columnar crystals	monoclinic (WO_3_) + orthorhombic (WO_3_·⅓H_2_O)	n.a.	n.a.
W7.6NaS	20 mL 0.1 M H_2_WO_4_ + SO_4_^2−^ (from Na_2_SO_4_)	7.6	1.15	nanobundles	hexagonal (WO_3_)	44.4	2.68
W0.3HS	20 mL 0.1 M H_2_WO_4_ + H_2_SO_4_	0.3	<0.3	nanocubes	monoclinic (WO_3_) + orthorhombic (WO_3_·⅓H_2_O)	n.a.	n.a.
W7.6HS	20 mL 0.1 M H_2_WO_4_ + H_2_SO_4_	7.6	<0.3	nanocubes	monoclinic (WO_3_) + orthorhombic (WO_3_·⅓H_2_O)	7.0	2.75
WSA	Commercial WO_3_ (Sigma Aldrich)	-	-	nanoparticles	monoclinic (WO_3_)	8.3	2.61

Using different amounts of the sulphate additive in hydrothermal synthesis can produce different WO_3_ crystalline phases. At the lower SO_4_^2−^/WO_4_^2−^ ratio (W0.3NaS), XRD indicated that the crystalline phase was the same as W0 (Table 1 and Supporting Information, Figure S1). The addition of excess sulphate additive induces a change in the crystalline phase from a mixture of monoclinic and orthorhombic in W0.3NaS to hexagonal-phase WO_3_ (cell constants: a = 7.3244 Å, b =7.3244 Å, c = 7.6628 Å; JCPDS 01-085-2459) in W7.6NaS. This observation suggests that not only does the sulphate additive play a crucial role in altering the morphology, but also in governing the crystalline phase of the product. Studies by Gu *et al.* [30] and Huang *et al.* [17] have also demonstrated the role of sulphate salt addition in determining crystalline phase of the WO_3_ product. Upon adding an increased amount of sulphate alkali metal salts, it was reported that the hexagonal phase becomes gradually dominant, which agrees with our result. Here, although the sodium ions in the Na_2_SO_4_ additive were removed during the ion exchange process, it is possible that a certain amount of Na^+^ ions still exist in the precursor solution (especially in W7.6NaS with a much higher Na_2_SO_4_ concentration) and therefore act as stabilising ions for the hexagonal and triangular tunnels in the formation of metastable hexagonal WO_3_.

To further investigate the unique behaviour of sulphate ions, H_2_SO_4_ was also used as a capping agent. The amount of H_2_SO_4_ added was adjusted, such that the SO_4_^2−^/WO_4_^2−^ ratios were equivalent to those used during W0.3NaS and W7.6NaS synthesis. A consequence of using H_2_SO_4_ was a simultaneous increase in the free H^+^ ions present, whereby the pH dropped to below 0.3. At this low pH, the resulting WO_3_ crystal morphology did not resemble any of the previous systems. Aggregated nanocubes (edge length of approximately 100–200 nm) were obtained for both W0.3HS and W7.6HS (Figure 1D,E, respectively), with the size of the WO_3_ particles being noticeably smaller than for the Na_2_SO_4_ additive. The diffraction lines for both powder samples (XRD spectra are located in Supporting Information, Figure S1) were designated as containing mainly monoclinic WO_3_ (cell constants: a = 7.2970 Å, b = 7.5390 Å, c = 7.6880 Å; JCPDS 01-071-2141) and partially orthorhombic WO_3_·⅓H_2_O (cell constants: a = 7.3590 Å, b = 12.5130 Å, c = 7.7040 Å; JCPDS 00-035-0270). It appears that the addition of more H_2_SO_4_ has little influence in terms of the overall morphology of the product or the crystalline phase, at least for SO_4_^2−^/WO_4_^2−^ ratios over the range 0.3 to 7.6.

It is suspected that the presence of the H^+^ ions is mainly responsible for both the cuboid morphology and the monoclinic-orthorhombic crystalline phase. pH has been demonstrated by Reis *et al.* [31] to have a marked influence on the crystalline phase of structures formed during hydrothermal acid hydrolysis of sodium tungstate (Na_2_WO_4_.2H_2_O), while Gu *et al.* [28] established pH variations over the range <0.5, 1.0–1.4 and 1.5–2.0 gave nanoparticle, nanobundle and nanowire WO_3_ structures, respectively. A possible explanation for the particle size shrinkage observed for the hydrothermally produced W0.3HS and W7.6HS samples might be that at a low pH value, the tungstic acid solution is so supersaturated that nucleation occurs homogeneously, rapidly generating a large number of small WO_3_ nuclei [32]. This nucleation reduces the supersaturation and consumes most of the resources for growth, which then results in a briefer ripening process.

Band-gaps derived from UV-Vis spectra (see Supporting Information, Figure S2), which are an indicator of the wavelength of light capable of photoactivating the material, ranged between 2.61 for WSA to 2.75 for W7.6HS (Table 1), which is typical for WO_3_ [24]. Table 1 also indicates that the specific surface area of the WO_3_ nanobundles is around five- to six-times greater than both the nanocubes and the nanoparticles.

### 2.2. Pt/WO_3_ Characteristics

**Figure 2:**
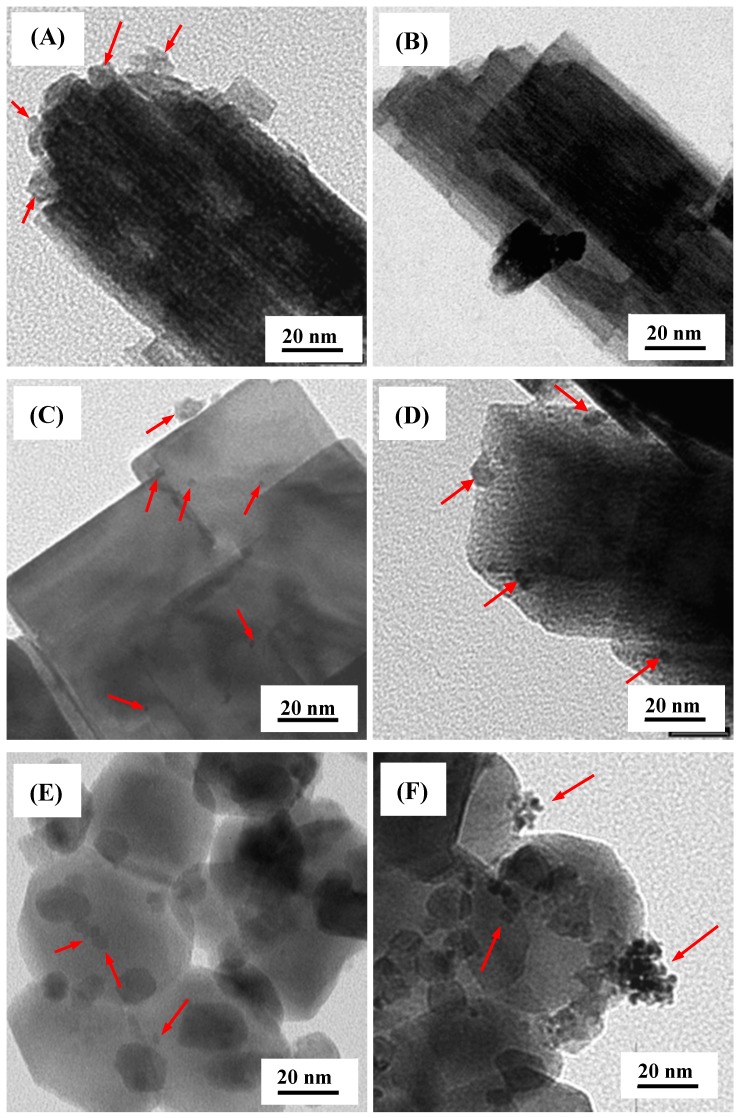
TEM micrographs of Pt photodeposited on WO_3_ nanorod bundles W7.6NaS using (**A**) UV-A light and (**B**) visible light; WO_3_ nanocubes W7.6HS using (**C**) UV-A light and (**D**) visible light; Sigma Aldrich WO_3_ WSA using (**E**) UV-A light and (**F**) visible light. Arrows indicate the Pt deposits.

TEM micrographs showing Pt deposits on the various particle morphologies are provided in Figure 2. Figure 2A suggests that for W7.6NaS, there are Pt deposits (with diameters over the range 5–10 nm) loaded onto the nanorods when using UV-A light, although similarities in contrast between the Pt and WO_3_ make them difficult to discern. Identifying Pt deposits on W7.6NaS following visible light photodeposition (Figure 2B) was challenging with no clear evidence of Pt deposits being present in the TEM image. The presence of Pt being photodeposited on the WO_3_ nanocubes (W7.6HS) using both UV-A and visible light was more apparent, as illustrated in Figure 2C,D, respectively. In both instances, the Pt deposits are hemispherical and dispersed in nature, with photodeposition under UV-A light appearing to give a greater prevalence of smaller (2–3 nm) deposits. Visible light photodeposition appears to favour Pt deposits in the range of 5–10 nm, although photodeposition using UV light also provided some larger Pt deposits (~10 nm). The WSA sample exhibited the most distinct difference in Pt photodeposit characteristics arising from the different light sources. Figure 2E indicates that Pt photodeposits from UV-A light are present as individual, roughly hemispherical deposits, although, again, the low contrast makes them difficult to identify. Visible light Pt photodeposition on WSA (Figure 2F) results in clusters of small Pt deposits accumulating on the WO_3_ particles. Pt nanoparticles within the clusters appear to have diameters of 2–3 nm, while the clusters themselves are up to 20 nm in diameter. It also appears that individual Pt deposits are present on the WO_3_ particles. From the TEM images, we can infer that the different WO_3_ morphologies and the light wavelengths impact the Pt photodeposition process. Particle morphology (e.g., nanocube *versus* irregular) will govern the number of potential sites (e.g., steps, edges, defects) where Pt deposition is favoured, as too will the crystalline phase (e.g. monoclinic *versus* hexagonal).

**Figure 3:**
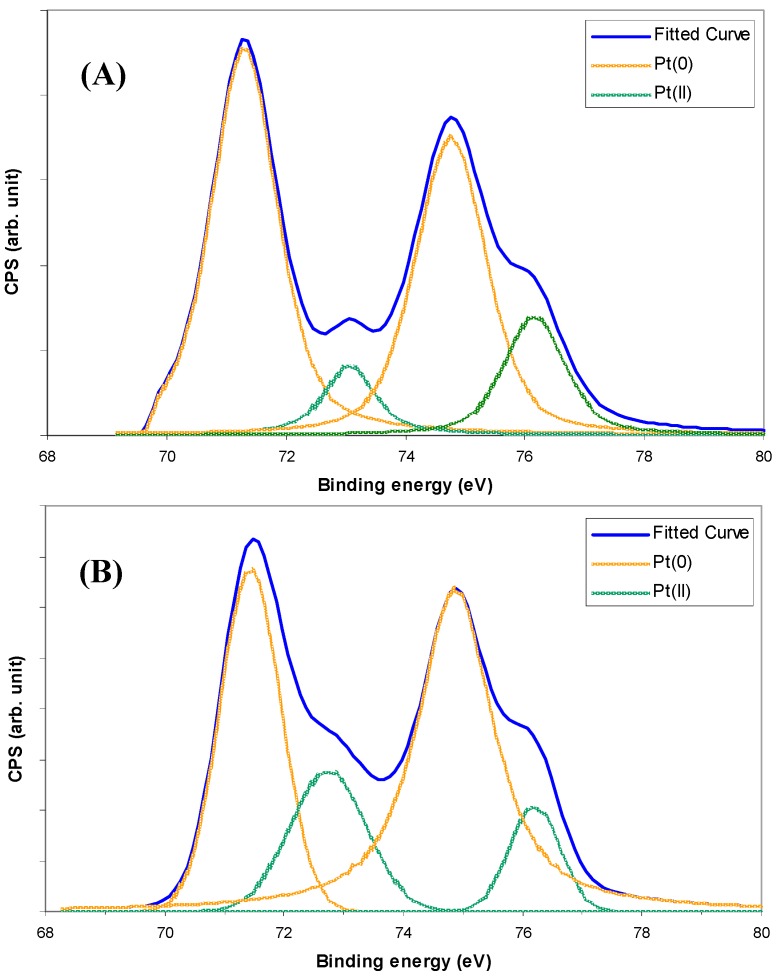
XPS spectra over the platinum Pt4f region for platinum photodeposited on WO_3_ nanocubes (W7.6HS) using: (**A**) UV-A light for 1 h; (**B**) visible light for 3 h.

The valence state of the deposited platinum nanoparticles plays an important role during charge trapping and interfacial charge transfer, and subsequently, the photocatalytic efficiency of the resulting platinised metal oxide. Figure 3 shows the XPS spectra of the platinised WO_3_ nanocuboids (W7.6HS) in the platinum (Pt4f) region (70–79 eV) arising from both UV-A and visible light photodeposition. In terms of the platinum oxidation state, the spectrum could be deconvoluted into two pairs of doublets. The main doublets located *ca*. 71.2 and 74.8 eV were attributed to atomic state Pt^0^, while the second pair of doublets at *ca*. 73.1 eV and 76.2 eV corresponded to the oxidation states of Pt^II^. By comparing the normalised peak areas, Pt^0^ was found to be the dominant species from both UV-A and visible light photodeposition. However, the relative percentage of Pt^II^ in the deposits was greater for the visible light particles (44%) than the UV-A particles (21%). The difference in the extent of reduced Pt between the two samples may arise from the higher tendency of Pt^IV^ to be reduced (Equation (5)) compared to Pt^II^ (Equation (4)) [33]. Although we have employed a longer irradiation time during the photodeposition of platinum on WO_3_ supports using visible light (3 h) compared to UV (1 h), we cannot exclude the possibility that the total amount of photons from the visible light illumination were not enough to reduce the Pt precursor into Pt metal.

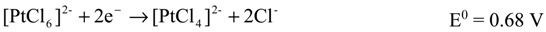
(4)

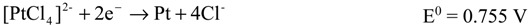
(5)

### 2.3. Ethylene Photodegradation

**Figure 4:**
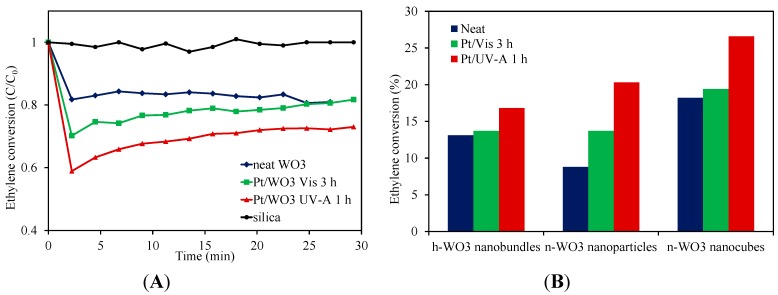
Photodegradation profiles displaying: (**A**) ethylene conversion with time for neat WO_3_ nanocubes (W7.6HS) and WO_3_ nanocubes platinised using visible light for 3 h or UV-A light for 1 h. Included is a silica control profile. (**B**) Steady-state ethylene conversion (as a percentage of initial ethylene concentration) for neat and platinised (visible light for 3 h or UV-A light for 1 h) WO_3_ nanobundles (W7.6NaS), nanoparticles (WSA) and nanocubes (W7.6HS).

Ethylene photodegradation profiles for neat WO_3_ nanocubes (W7.6HS) and WO_3_ nanocubes loaded with Pt using either visible light (3 h) or UV-A (1 h) are provided in Figure 4A. Upon illumination, the ethylene concentration drops sharply within the first 2.5 min for all samples, after which it increases slowly, eventually reaching a stable conversion level. The gradual increase in the ethylene concentration after the initial drop was postulated to be due to the formation of intermediate products, which compete with ethylene during the photocatalytic degradation process. Unfortunately, these intermediate products were not able to be detected by the GC/flame ionisation detector (FID) instrument, such that no identification of these products was available. Once stabilised, ethylene conversion was in the order neat WO_3_ ≈ Pt/WO_3_ (visible light 3 h) < Pt/WO_3_ (UV-A light 1 h). Despite displaying similar ethylene conversion levels after 30 min of illumination, it appears that, at least initially, the WO_3_ nanocubes platinised using visible light are more active than the neat WO_3_ nanocubes. The findings indicate that loading platinum deposits on the WO_3_ nanocubes improves their capacity for photodegrading ethylene. The greater photoactivity exhibited by the nanocubes loaded with Pt using UV-A light may arise from the greater portion of Pt^0^ within the deposits (Figure 3). Pt^0^/TiO_2_ has been reported to be more active than Pt^II^/Pt^IV^ species on TiO_2_ for photocatalytic degradation of various organic contaminants [34,35]. The Pt^II^ or Pt^IV^ can undergo consecutive oxidation/reduction cycles, which consume excited charge carriers and lowers performance. A control system comprising only the washed silica beads is included in Figure 4 for comparison showing that the ethylene photodegradation derives solely from the neat and platinised WO_3_ nanostructures.

Figure 4B demonstrates that particle morphology has an influence on photocatalytic performance. In the case of neat WO_3_, the activity for ethylene photodegradation was in the order of nanocubes > nanobundles > nanoparticles. Despite having similar surface areas (Table 1), the WO_3_ nanocubes exhibit approximately 10% greater conversion of ethylene over the nanoparticles once the photoactivities had stabilised. This may be due to the more “edged” nature of the cuboid morphology, reducing the photogenerated electron-hole recombination, similar to that described by Kato *et al.* [36] in their study on photocatalytic water splitting by NiO/NaTaO_3_. Alternately, the relative proportion of different exposed crystal facets for the different morphologies may have influenced the photoactivity, as was demonstrated by Xie *et al.* for WO_3_ [37]. They observed that WO_3_ in a sheet-like form possessed a greater percentage of the (002) facet compared with WO_3_ cuboids. The dominance of the (002) facet blue-shifted the band-gap of the sheet-like structure, as well as increased the reduction potential of the conduction (and valence) band(s), relative to the cuboid structure. The deeper valence band maximum of the cuboid structure was used to account for it being the more effective structure when evolving oxygen during photocatalytic water oxidation in the presence of an electron acceptor. Interestingly, the nanobundles have a comparably higher surface area than the other nanostructures and a partially edged morphology, but do not display a correspondingly larger photoactivity. This may result from the hexagonal crystalline phase not being as active as the monoclinic-orthorhombic phase, with this postulation requiring further investigation.

Platinising the WO_3_ nanoparticles and nanocubes appears to have a more pronounced effect on ethylene photodegradation than for the nanobundles, especially when UV-A is used as the light source. In this instance, the photoactivity order becomes Pt/nanocubes > Pt/nanoparticles > Pt/nanobundles. This could derive from the hexagonal crystalline phase of the nanobundles being less active than the monoclinic phase and/or the comparative lack of Pt deposits on the nanobundles. Further study is needed to identify the parameter responsible for the result.

**Figure 5:**
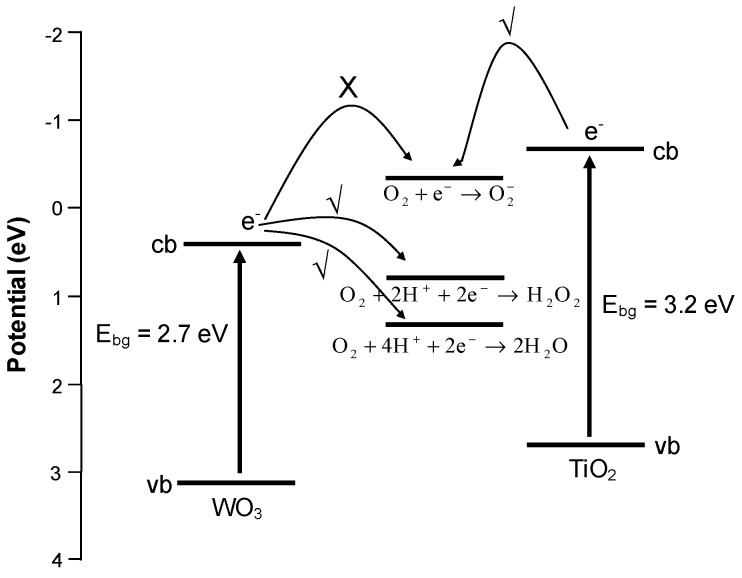
Valence and conduction band potentials for WO_3_ and single and multi-electron potentials for oxygen. The valence and conduction band potentials for TiO_2_ are included for comparison.

In general, the presence of metal deposits on the surface of a photocatalyst acts as an electron sink, trapping electrons and allowing for greater photogenerated hole availability for photodegradation reactions. However, the Pt deposits can also facilitate the transfer of electrons to electron scavengers in the system, most often O_2_ (Equation (6)). Wang *et al.* [38] suggested that the consumption of electrons by O_2_ is the rate limiting step in photocatalysis, and the presence of metal deposits can help alleviate this. In the instance of WO_3_, the reduction potential for the photogenerated electron (present in the conduction band) is not large enough for the single electron reduction of O_2_. That is, it does not possess enough energy to be picked up by O_2_ in accordance with Equation (6). However, the photogenerated electron does have enough energy to participate in the multi-electron reduction of O_2_ (Equations (7) and (8)), but the need for more than one electron in close proximity to invoke this reduction is not as favourable. This is depicted schematically in Figure 5. The valence and conduction band potentials of TiO_2_ are also provided for comparison.

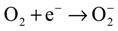
(6)

O_2_ + 2H^+^ + 2e^−^ → H_2_O_2_ (7)

O_2_ + 4H^+^ + 2e^−^ → 2H_2_O_2_ (8)

The presence of Pt facilitates the multi-electron reduction of O_2_ due to the ready availability of electrons in close proximity to one another on the Pt deposit. Consequently, the Pt reduces the limitation of the smaller conduction band electron potential of WO_3_, in turn promoting the photocatalytic activity. Similar to the result from this study, Abe *et al.* [21] also found that the decomposition of organic compounds under visible light irradiation with platinum loaded WO_3_ sample was enhanced significantly and postulated this to be due to the promotion of multi-electron O_2_ reduction on the Pt co-catalyst.

## 3. Experimental Section

### 3.1. Materials

Commercial tungsten (VI) oxide nanopowder, WO_3_ (Sigma-Aldrich, St. Louis, MO, USA, denoted as “WSA”), with a mean particle size of less than 100 nm was used as the benchmark photocatalyst. For the hydrothermal synthesis of WO_3_ nanostructures, the following chemicals were used as received: Na_2_WO_4_·2H_2_O (99.0%, A.C.S. reagent, Sigma-Aldrich); Na_2_SO_4_, (99.0%, Ajax Finechem Pty. Ltd., Auburn, NSW, Australia) and “Amberlite” IR-120 (H) ion-exchange resin (BDH Laboratory Supplies).

Platinum (Pt) was loaded onto the WO_3_ nanostructures by a photodeposition method. H_2_PtCl_6_ (Sigma-Aldrich) was employed as the platinum precursor, and methanol, CH_3_OH (99.7%, Ajax Finechem Pty. Ltd.), acted as the hole-scavenger in the system. Nitrogen gas, N_2_ (>99%, Coregas Pty. Ltd., Yennora, NSW, Australia), was used to purge the solution during the photodeposition reaction.

Visible light photodegradation focused on ethylene (508 ppm, Coregas Pty. Ltd.) as the VOC model pollutant. Compressed air (79% N_2_, 21% O_2_, Coregas, Pty. Ltd.) acted as the diluent of the gas stream, while compressed nitrogen, N_2_ (>99%, Coregas Pty. Ltd.), was used as the carrier gas in the gas chromatograph. The flame ionisation detector (FID) was fuelled by compressed hydrogen, H_2_ (Coregas, Pty. Ltd.), blended with compressed air.

### 3.2. Hydrothermal Synthesis of WO_3_ Nanostructures

Five variants of WO_3_ particles were hydrothermally synthesised: one without any shape-directing agent, denoted as “W0” (where “W” represents tungsten trioxide and “0” represents no shape directing agents); two with different concentrations of Na_2_SO_4_, denoted as “W_x_NaS” (where “x” represents the SO_4_^2−^:WO_4_^2−^ molar ratio during hydrothermal synthesis and “NaS” indicates that Na_2_SO_4_ was the shape directing agent); and two with different concentrations of H_2_SO_4_, denoted as “W_x_HS” (where “HS” indicates that H_2_SO_4_ was the shape directing agent). An ion-exchange column containing approximately 35 g of “Amberlite” IR-120 (H) was set up to remove sodium ions (Na^+^) from the NaWO_4_ solution. When preparing the precursor for W0 and W_x_HS synthesis, a solution of Na_2_WO_4_·2H_2_O (0.1 M) was passed through the column at 1 mL/min, and the eluent was collected. The resulting tungstic acid (H_2_WO_4_·2H_2_O) solution displayed a pale yellow colour and possessed a pH between 1.61 and 1.67. In the case of the W_x_NaS precursor, a specified amount of Na_2_SO_4_ (see Table 1) was added to the H_2_WO_4_·2H_2_O (0.1 M) solution and then passed through the ion-exchange column to remove the Na ions from the Na_2_SO_4_.

To prepare the W0 particles, 20 mL of the H_2_WO_4_ precursor solution was initially placed in a 50-mL Teflon tube. In the case of W_x_HS particle synthesis, a small amount of H_2_SO_4_ with a specified concentration (see Table 1) was added to the H_2_WO_4_ precursor solution in the Teflon tube. For W_x_NaS particle synthesis, 20 mL of the mixed tungstic-sulphate precursor was placed in the 50-mL Teflon tube. Following precursor addition, the Teflon tube was then sealed in a stainless steel autoclave and heated in an oven at 200 °C for 10 h. The resulting precipitate was recovered by centrifuging (Beckmann Coulter Allegra 25R, 10,000 rpm), washed at least five times with deionised water to remove any unreacted precursors and air-dried in a 60 °C oven for approximately 15 h.

### 3.3. Photodeposition of Platinum on WO_3_ Nanostructured Supports

Platinum was loaded onto the WO_3_ nanostructure surface using photodeposition in a 500-mL Pyrex glass annular reactor surrounding a 20-W NEC T10 black light blue lamp (λ_max_ = 360 nm). A 1 g/L WO_3_ slurry was dispersed ultrasonically for 15 min, with 550 mL of this slurry transferred to the photoreactor and circulated for 30 min under illuminated conditions. Light pre-treatment was designed to remove adsorbed organic impurities from the particle surface. The UV lamp was then turned off, and 2000 µg carbon, in the form of methanol, were added to the system as a hole scavenger. The metal precursor (H_2_PtCl_6_) was added to give the required Pt loading (1.0 at. %); the pH was adjusted to 3 using dilute perchloric acid, and the system was purged with nitrogen gas at a flow rate of 50 mL/min for 20 min. The conditioned slurry was illuminated for 60 min, recovered by centrifuging and washed with deionised water for a minimum of five times. The washed particles were dried in an oven at 60 °C for 12 h and then ground and stored in a desiccator prior to use.

As a comparison, the effect of using visible light as the source of illumination during Pt photodeposition was investigated. In this instance, an 18-W fluorescent light (Sylvania Luxline Plus F18/860, Erlangen, Germany) with a cut-off filter (λ > 420 nm) was employed as the light source. The photodeposition procedure was the same as for the UV-lamp apart from the illumination period (during Pt photodeposition) being extended to 3 h, due to the weaker irradiance of the fluorescent lamp.

### 3.4. Photocatalytic Oxidation of Ethylene

Neat and Pt-loaded WO_3_ nanostructures were assessed for gas-phase ethylene photodegradation using an annular-type packed-bed photoreactor, as described previously [39]. The photoreactor consisted of a 400 mm-long Pyrex glass tube (15 mm outside diameter (o.d.), 1.2 mm wall thickness) containing a glass filler tube (12 mm o.d.). The packed-bed reactor was prepared by filling the annular gap with a catalyst/silica bead mixture in a 1:10 ratio (total weight of 0.66 g) to give a catalyst bed approximately 50 mm in length. The bed was supported at each end by washed silica beads and held in position by quartz wool. Prior to use, the silica beads were cleaned with 1 wt % HCl solution and then rinsed with deionised water until no pH change was observed. The washed beads were then dried in an oven at 100 °C. Illumination was provided by four 6-W Sylvania fluorescent lamps spaced equidistantly around the reactor. Inlet ethylene concentration was maintained at 50 ppm in air at a flow rate of 20 mL/min. Ethylene concentration was measured using a Shimadzu GC-8A (Shimadzu Co., Tokyo, Japan) equipped with an FID. Gas component separation was achieved with an Alltech HAYESEP Q 80/100 column (Alltech Associates, Inc., Deerfield, IL, USA).

Photocatalysis experiments involved passing 10 mL/min air through the packed bed for 10 min, whereby the lamps were switched on for 60 min to remove impurities from the photocatalyst surface. The lamps were then switched off, and the ethylene:air mix passed through the bed (20 mL/min) until ethylene concentration in the reactor effluent was the same as that in the reactor inlet. At this point, the lamps were switched on and the reactor effluent analysed for ethylene concentration. Samples were taken every 2 min.

### 3.5. Characterisation

Morphology characterisation of the samples was obtained by a scanning electron microscope (Hitachi S900 SEM, Tokyo, Japan) at an applied voltage of 4 kV and a high resolution transmission electron microscope (CM200 TEM, Philips Co., Amsterdam, The Netherlands) operated at 200 kV. X-ray diffraction (XRD) spectroscopy with a Philips X’pert Pro MPD (Philips Co., Eindhoven, The Netherlands), Cu Kα1 radiation λ = 1.54060 Å, 45 kV, 40 mA, was used to identify the crystalline phase of the product. N_2_ physisorption on a Micromeritics TriStar 3000 (Micromeritics, Norcross, GA, USA) was employed to evaluate the specific surface area of the product. Prior to analysis, the sample was degassed at 150 °C under vacuum overnight. The Brunauer–Emmett–Teller (BET) model (5-points) was used to determine the specific surface area. The photoresponses and band gap properties of all tungsten oxide catalysts were measured using UV-Vis spectroscopy (Varian Cary 300) from 200–800 nm, with barium sulphate (BaSO_4_) as the reference material. The oxidation states of platinum nanodeposits were determined by X-ray photoelectron spectroscopy (XPS-EscaLab 220-iXL, Al Kα radiation (1486.6 eV), Thermo VG Scientific Ltd., East Grinstead, UK). Inductively coupled plasma-atomic emission spectroscopy (ICP-AES, Varian Vista AX, Varian, Palo Alto, CA, USA) was used to determine the Na content within the synthesised particles.

## 4. Conclusions

The presence of SO_4_^2−^ anions and pH control has been demonstrated to play an important role in controlling the final morphology and crystalline phase of hydrothermally synthesised WO_3_ nanostructures. SO_4_^2−^ anions promoted the formation of hexagonal nanobundles, while at pH values below 0.3, nanocube formation with a monoclinic-orthorhombic crystalline structure was favoured. The influence of WO_3_ nanostructure morphology and crystalline phase on its capacity to photodegrade ethylene using visible light was investigated. The WO_3_ nanocubes provide the best photodegradation performance due to their unique geometric configuration. The presence of Pt deposits improved the photoactivity of the nanoparticles and nanocubes, which was assigned to the Pt deposits ability to facilitate the multi-electron reduction of O_2_. Utilising different light sources (*i.e*., visible light or UV-A) to photodeposit the Pt on the WO_3_ nanostructures altered the Pt deposit morphology, size and oxidation state, which also influenced the photocatalytic performance. The smallest photoactivity improvement was apparent for Pt loaded on the nanobundles, with this tentatively attributed to their hexagonal crystalline phase.

## Supplementary Materials

Supplementary materials can be accessed at: http://www.mdpi.com/1420-3049/19/11/17747/s1.

## Supplementary Files

Supplementary File 1
