# Supplementary material for: Tungsten Trioxide as a Visible Light Photocatalyst for Volatile Organic Carbon Removal
Source: Molecules. 2014 Oct 31;19(11):17747–62. doi: 10.3390/molecules191117747 (PMC6271203; doi:10.3390/molecules191117747)

Supplementary Materials

Figure S1. XRD spectra for the different WO_3 photocatalysts: (**A**) Hydrothermally synthesised WO_3 with no additives (W0); (**B**) Hydrothermally synthesised WO_3 with Na_2SO_4 added at a SO_4^{2-}:WO_4^{2-} ratio of 0.3 (W0.3NaS); (**C**) Hydrothermally synthesised WO_3 with Na_2SO_4 added at a SO_4^{2-}:WO_4^{2-} ratio of 7.6 (W7.6NaS); (**D**) Hydrothermally synthesised WO_3 with H_2SO_4 added at a SO_4^{2-}:WO_4^{2-} ratio of 0.3 (W0.3HS); (**E**) Hydrothermally synthesised WO_3 with H_2SO_4 added at a SO_4^{2-}:WO_4^{2-} ratio of 7.6 (W7.6HS); (**F**) Commercial Sigma Aldrich WO_3 (WSA). m = monoclinic (WO_3), o = orthogonal ($WO_3 \cdot ⅓H_2O$) and h = hexagonal (WO_3).

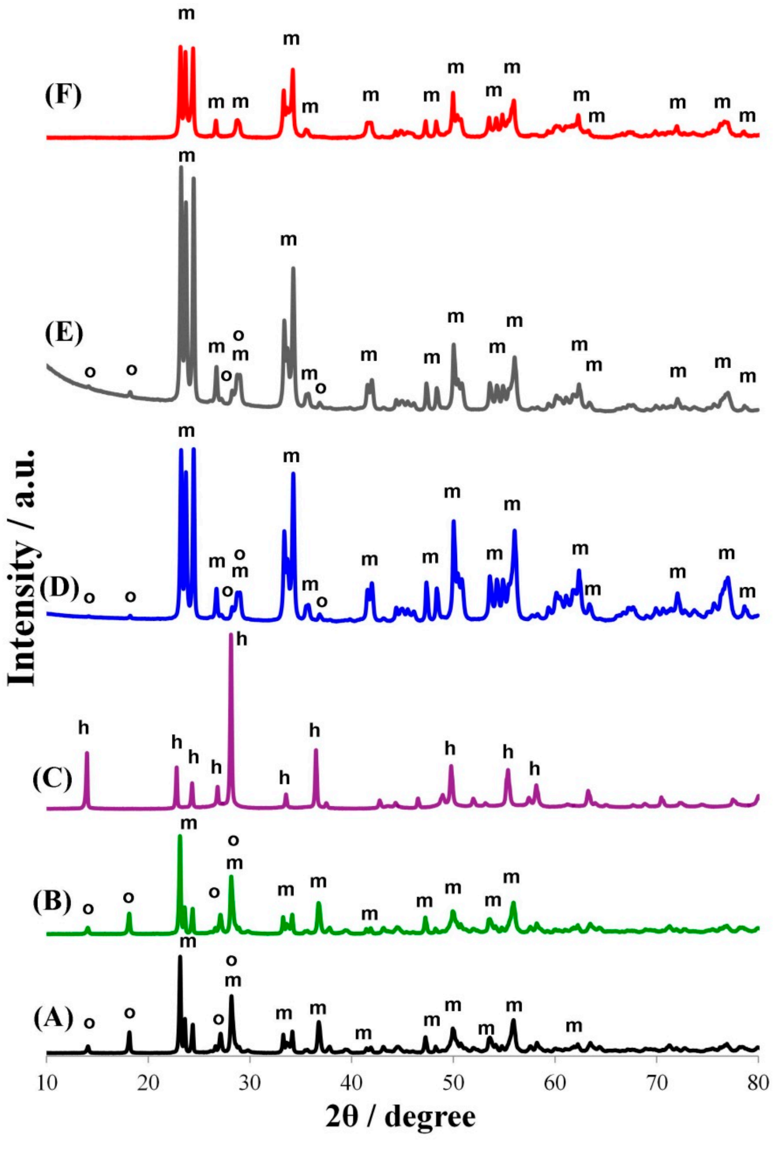

Figure S2. Modified Kubelka–Munk Plot of neat WO_3 nanocubes (W7.6HS), Sigma Aldrich WO_3 (WSA) and WO_3 nanorod bundles (W7.6NaS).

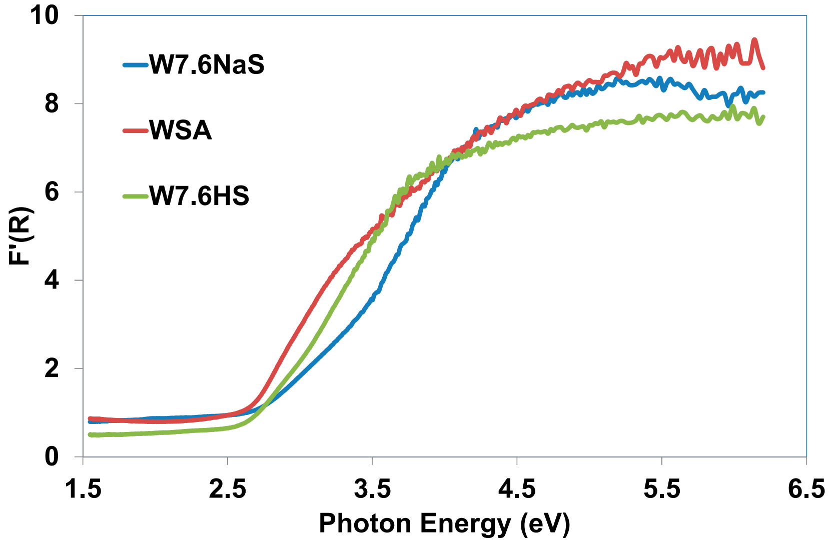

Supplement: Supplementary File 1 [file molecules-19-17747-s001.pdf]
